# Privacy protection framework for face recognition in edge-based Internet of Things

**DOI:** 10.1007/s10586-022-03808-8

**Published:** 2022-11-17

**Authors:** Yun Xie, Peng Li, Nadia Nedjah, Brij B. Gupta, David Taniar, Jindan Zhang

**Affiliations:** 1 School of Computer Science, Nanjing University of Posts and Telecommunications, Nanjing, 210023 China; 2 Department of Electronics Engineering and Telecommunications of the Engineering Faculty, State University of Rio de Janeiro, Rio de Janeiro, Brazil; 3 International Center for AI and Cyber Security Research and Innovations & Department of Computer Science and Information Engineering, Asia University, Taichung, Taiwan; 4 Center for Interdisciplinary Research, University of Petroleum and Energy Studies (UPES), Dehradun, India; 5 Lebanese American University, Beirut, Lebanon; 6 Faculty of Information Technology, Monash University, Clayton, Australia; 7 Xianyang Vocational Technical College, Xianyang, China

**Keywords:** Face recognition, Eigenface, Local differential privacy, Edge computing

## Abstract

Edge computing (EC) gets the Internet of Things (IoT)-based face recognition systems out of trouble caused by limited storage and computing resources of local or mobile terminals. However, data privacy leak remains a concerning problem. Previous studies only focused on some stages of face data processing, while this study focuses on the privacy protection of face data throughout its entire life cycle. Therefore, we propose a general privacy protection framework for edge-based face recognition (EFR) systems. To protect the privacy of face images and training models transmitted between edges and the remote cloud, we design a local differential privacy (LDP) algorithm based on the proportion difference of feature information. In addition, we also introduced identity authentication and hash technology to ensure the legitimacy of the terminal device and the integrity of the face image in the data acquisition phase. Theoretical analysis proves the rationality and feasibility of the scheme. Compared with the non-privacy protection situation and the equal privacy budget allocation method, our method achieves the best balance between availability and privacy protection in the numerical experiment.

## Introduction

The advantages of FR technology in terms of non-contact and convenience have applied themselves to a greater extent in applications such as health testing, mobile payment, personal information collection, and identity authentication, especially since the outbreak of COVID-19. The vigorous development of machine learning (ML) [1, 2] has greatly improved the accuracy and performance of FR in the era of big data, far surpassing traditional methods [3, 4]. However, the constraints caused by limited local storage and computing resources still exist, especially when dealing with large-scale face databases [5].

EC solves the above difficulties in a new way. It analyzes and processes data near the source of data, and there is no data circulation [6]. In EFR systems, face data still needs to be submitted to an untrusted third-party cloud server for model training after edge processing [7]. The issue of privacy cannot be overlooked because face data carries or is closely related to personal sensitivities information [8]. Based on these concerns, federal agencies in the United States [9] have tried to promote new regulations to protect privacy, that is, banning FR systems.

Therefore, privacy protection is still a significant issue in face recognition systems in edge environments. Privacy protection for EFR systems faces several core issues:

- Do not rely on the existence of a trusted third-party server;
- The attacker cannot associate the acquired facial features with other sensitive data;
- Facial biometrics should be irreversible one-way conversion;
- Computational complexity shows friendliness to resource-constrained equipment and can be extended to large-scale data processing occasions.

Starting with secure data transmission and access control is the most traditional method to solve the privacy protection problem of the FR system, for example, ML frameworks based on homomorphic encryption technology, SecureML [10], and DeepZeroID [11]. In addition, although the loss of FR accuracy caused by encryption technology is tiny, its high time complexity and large memory consumption are not suitable for practical applications. Compared with traditional methods, differential privacy (DP) achieves lower computational complexity at the expense of proper utility. Especially in large-scale data processing, DP better reflects high efficiency [12].

Besides that, most FR functions based on encryption methods that provide privacy protection require an assumption that there exits one trusted third party [13, 14] in the server-based settings. However, there is no fully trusted party in the actual scenario. Generally, after the cloud server receives the encrypted data, it decrypts to get the plaintext and then performs matching and analysis operations [15]. There is a high possibility that private data leak on the server-side.

On the contrary, DP technology applies to the authentic situation where an untrusted third party exists, which has become a new focus in FR [16]. However, most existing schemes based on DP only consider data privacy protection in one or several stages of storage, release, and model construction in EFR systems [17]. There is still a lack of a systematic approach to address all the core issues mentioned above. For these reasons, this paper focuses on privacy protection throughout the entire life cycle of data in EFR systems. The main contributions of our work are summarized as follows:

- We propose a general privacy protection framework for an EFR system, in which the terminal device, the edge network center, and the remote cloud server construct a three-level FR architecture. The privacy protection of face data in the entire life cycle is focused on and realized.
- We design a LDP algorithm that adaptively allocates a privacy budget according to the difference in the proportion of principal component feature information. The edge executes this algorithm after the dimensionality reduction of face images, which, on the one hand, protects the face feature data transmitted between edges and the cloud, and on the other hand, enhances the privacy security of the stored data and the published model on the cloud side.
- We add an authentication mechanism to control the legitimacy of terminal devices connected to the edge network center for higher security. This mechanism ensures the reliability and quality of the data source.

The rest of this article is organized as follows: Sect. 2 discusses related work, and Sect. 3 provides background knowledge. The EFR system and its existing security threats are explained in Sect. 4. Section 5 elaborates on the privacy protection scheme in detail. Section 6 discusses the experimental process and analyzes the results. Finally, Sect. 7 concludes the article.

## Related work

Typical privacy protection methods in FR include encryption, de-identification (de-ID), and perturbation. We review the most relevant related works by category as follows.

### Encryption for FR

Homomorphic encryption and secure multi-party computing are essentially encryption. In 2009, Erkin et al. [13] proposed a privacy-preserving FR scheme using Paillier and DGK cryptosystem. Since the recognition of the face image in the database is to process the homomorphically encrypted data, the computational complexity is very high. Sadeghi et al. [18] subsequently proposed a relatively effective improvement method. That is pulling in an obfuscation circuit in homomorphic encryption to improve the recognition efficiency. Xiang et al. [14] proposed a hybrid encryption scheme based on fully homomorphic for the scenario of FR outsourcing to cloud servers. The protocol provided higher privacy for faces and reduced the computational cost between the user and the face owner.

Compared with homomorphic encryption technology, secure multi-party computing technology is more suitable for face data privacy protection in application scenarios with multiple cloud servers [19–21]. In [19], the authors presented an application of FR technology based on secure multi-party computing in CloudID and designed a *K*-d tree structure processing biometrics in the encrypted domain. In [20], a secure outsourcing FR method for the joint-environment has been given, which can still effectively protect the user’s private data under the semi-honest model. The main idea is that two semi-honest and conflict-free cloud servers perform FR based on eigenface algorithms in a privacy-protecting manner. Ma et al. [21] also proposed a secure FR system, in which a deep neural network is for extracting facial features. To reduce the computational burden, lightweight computing schemes have been proposed: POR [22] and PE-MIU [23]. POR is a lightweight-adaptive enhancement AdaBoost classification framework based on additive secret sharing. PE-MIU implements privacy enhancement based on the smallest information unit.

### De-ID for FR

The de-ID method realizes privacy protection by removing the correspondence between the user’s face data and individual identities. *K*-same based on *K*-anonymity is an early method [24]. Then, some new de-ID methods appeared. Binod et al. [25] proposed a method to de-identify the face images by adding designed noise patterns. Letournel et al. [26] proposed an adaptive filtering method to de-identify face images. Sun et al. [27] focused on diversity to avoid de-IDed faces all looking similar. More recently, generative adversarial networks (GAN) have been used for face de-ID. The work in [28] used GAN to generate de-IDed faces. In [29], a GAN-based in-painting method was used to partially replace the original face.

### Perturbation for FR

Early face image privacy protection based on DP technology mainly solved the privacy protection problem during publishing face data by confusing the visual identity in the image. For example, Othman and Ross [30] interfered with the face image to hide age, gender, and race information. Although adding Laplace noise directly to all values in the real field matrix of the image can satisfy DP, it will cause excessive distortion of the images. To reduce the noise error, Zhang et al. [16] proposed an image compression method based on discrete Fourier transform, adding the corresponding Laplacian noise to the compressed image. However, reconstruction error is incited in the image compression process. To balance the noise error and the reconstruction error, they proposed an improved scheme based on matrix decomposition, which improved the robustness of the gray scale face image [31] but failed to fundamentally eliminate the reconstruction error in the compression process. In [32], Fan added pixelation to face images based on DP to achieve safe sharing while obtaining strong privacy guarantees. However, the confusing images are no longer similar to the original category of the object, resulting in poor visual quality of the output image. William et al. [33] applied DP to a generative model to blur facial images, and then extended this method to general images [34]. Liu et al. [35] adopted a data stream method to process images and finally realized the dynamic allocation of privacy budget based on the similarity of adjacent data, which improves the image data to a certain extent availability.

However, the above ways tend to focus on the research and application of theoretical methods in the scenes of trusting a third-party. In actual applications, third parties are usually untrustworthy. In response to this problem, Chamikara et al. [17] proposed the PEEP protocol to achieve resistance to member reasoning attacks and model memory attacks, in which the third-party server only receives and stores the disturbed eigenface data to perform a standard recognition algorithm.

## Preliminaries

### Local differential privacy

#### Definition 1

($\varepsilon$-LDP) [36] Assuming there are *N* users, each user has a data record. For privacy algorithm *F*, its defined domain is *Dom*(*F*), and its output range is *Ran*(*F*). If the same output result $t^{*}(t^{*}\subseteq Ran(F))$ which is obtained by the algorithm *F* on two arbitrary records *t* and $t^{\prime }( t,t^{\prime }\subseteq Dom(F))$ satisfies the condition of formula (1). Then we say that the algorithm *F* satisfies $\varepsilon$-LDP.

1 $$\begin{aligned} \Pr [F(t) = {t^*}] \le {e^\varepsilon } \times \Pr [F\left( {t^{\prime }} \right) = {t^*}] \end{aligned}$$

LDP is a branch of the $\varepsilon$-DP framework. LDP does not rely on a trusted data collector, and its privacy protection occurs on the user side. Each user submits only the disturbed data to an aggregator. The aggregator cannot distinguish whether $t^{*}$ is from the real record *t* or another record $t^{\prime }$ with high confidence, regardless of the background information it possesses. Even if the aggregator is malicious, the privacy of each user is still protected. Moreover, since the data is randomly disturbed at each user, users can use DP parameter values to achieve personalized privacy protection based on their own needs.

LDP inherits important properties of DP: such as post-processing immunity, which states that arbitrarily transforming the output of DP by some data-independent functions will not affect its privacy guarantee.

#### Theorem 1

(Post-processing) [37] Let *M* be an $\varepsilon$-differentially private mechanism and *g* be an arbitrary mapping from the set of possible outputs of *M* to an arbitrary set. Then, $g \circ M$ is $\varepsilon$-differentially private.

### Principal component analysis (PCA)

The PCA method is especially useful when the variables in the dataset are highly correlated, reducing the original variables to a smaller number of new variables (principal components). That is, PCA removes variables that are strongly correlated with other variables, leaving more representative variables. Compared with LDA and a few other dimensionality reduction methods [38], PCA can retain the original information to the greatest extent and minimize the loss of information while reducing the features.

Suppose the data set $D=[D_{1},D_{2},\ldots ,D_{n}]$ has a total of *n* samples, and each sample, such as the *i*th sample $D_{i}\in R^{d}$ , contains *d* characteristic attributes, then the original data set $D\in R^{n\times d}$ can be represented in a $n\times d$ matrix form.

#### Definition 2

(Covariance matrix (CM)) Assuming that the $l_{2}$ norm of the vector $D_{i}$ satisfies the condition: $\Vert D_{i}\Vert _{2}\le 1$ , the $l_{2}$ norm of any vector $x\in R^{d}$ is ${\left\| x \right\| _2} = \sqrt{\sum \limits _{i = 1}^d {{x_i}^2} }$. The CM of the original data $D=[D_{1},D_{2},\ldots ,D_{n}]$ is:

2 $$\begin{aligned} A = \frac{1}{n}{D^T}D = \frac{1}{n}\sum \limits _{i = 1}^d {{D_i}^T{D_i}} \end{aligned}$$

Here, *A* is a $d\times d$ symmetric matrix. *A* represents the correlation between variables, and the correlation indicates that there is redundancy in the data.

#### Definition 3

(Eigenvalue decomposition) The relationship among CM *A*, eigenvalue $\lambda$ and eigenvector *U* satisfies:

3 $$\begin{aligned} A = U\Lambda {U^T} \end{aligned}$$

Among them, $\Lambda$ is a diagonal matrix composed of $A^{\prime }$s all eigenvalues; *U* is an orthogonal matrix composed of $A^{\prime }$s all eigenvectors in columns, which constitutes a new vector space as the coordinate axis of new variables (principal components). Principal components can be obtained by calculating the eigenvalue $\lambda$ and the corresponding eigenvector *U* of *A*.

Start with the order polynomial of the eigenvalue $\lambda$:

4 $$\begin{aligned} \mid {\lambda I - A} \mid = 0 \end{aligned}$$

Among them, *I* is an identity matrix, the eigenvalue $\lambda$ of *A* are solved, and then by the formula:

5 $$\begin{aligned} A{u_i} = {\lambda _i}{u_i},(i = 1,2,...,d) \end{aligned}$$

Eigenvectors of *A* can be solved. Denote the set of eigenvectors as $U = ({u_1},{u_2}, \ldots ,{u_d})$. Here, $\lambda _{i} (1\le i\le d)$ is the *i*th eigenvalue corresponding the *i*th eigenvector $u_i$, which can represent the proportion of the information contained in the corresponding component, the larger the value $\lambda _{i}$, the more important the information contained in the corresponding component.

#### Definition 4

(Accumulative contribution) [39] Accumulative Contribution is used to reflect the proportion of information contained in the first *k* eigenvalues when the number of principal components is selected as *k*, expressed as:

6 $$\begin{aligned} {\eta _k} = {{{\sum \limits _{i = 1}^k {{\lambda _i}} }} \bigg / {{\sum \limits _{i = 1}^d {{\lambda _i}} }}} \end{aligned}$$

Introduce $\eta (0\le \eta \le 1)$ as the threshold, and make $\eta _{k} \ge \eta$ to determine the appropriate value of *k*. Then, extract the eigenvectors corresponding to the first *k* eigenvalues to form a matrix ${U_k} = ({u_1},{u_2},...,{u_k})$, in which $u_{i}$ and $u_{j}$ are orthogonal $(i\ne j)$.

## System framework

This section introduces the architecture of the EFR system and analyzes its existing security issues and design goals.

### System model

**Fig. 1:**
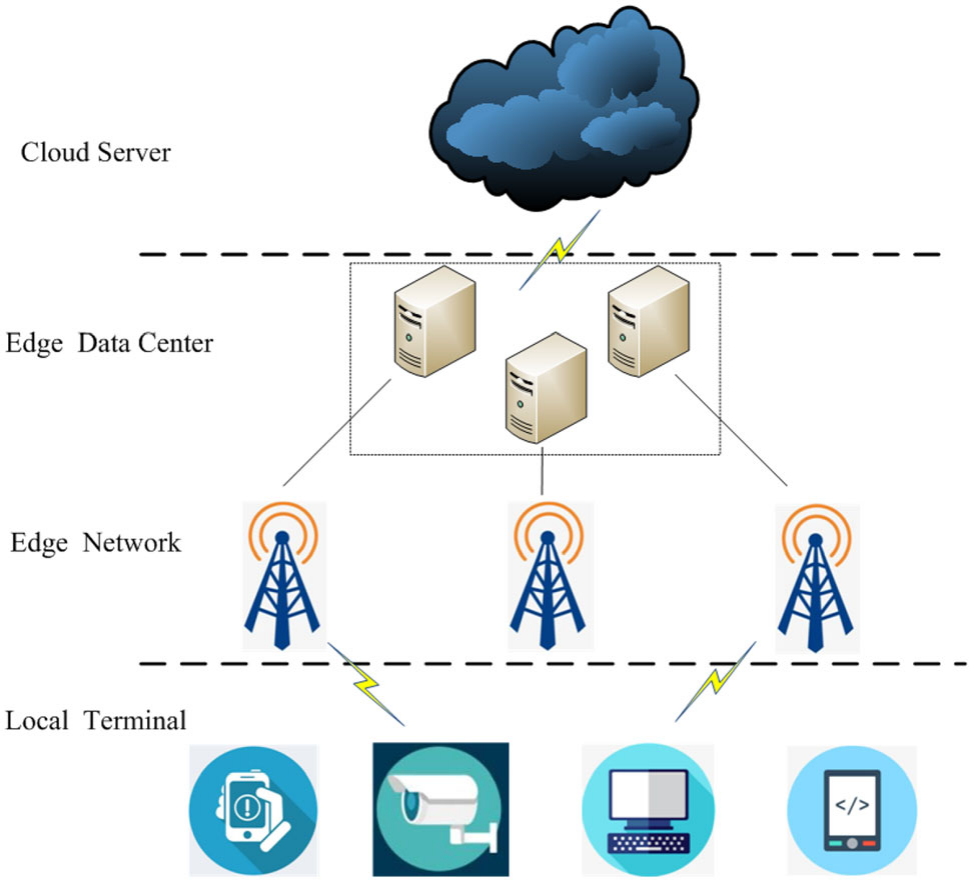
The architecture of EFR system

The EFR system framework is divided into three layers according to functions, as shown in Fig. 1.

- The first layer is the local terminals, which collect face images and uploads them to the edge.
- The second layer is the edge, which receives the face image uploaded by the local terminal and uploads it to the server after preprocessing.
- The third layer is the cloud server, which receives the face image information submitted by the edge, carries out model training, or uses the trained model for recognition, and feeds back the recognition result to the edge.

This framework, on the one hand, can use the existing data and computing capabilities on the cloud server to complete model training; on the other hand, it reduces the pressure of data transmission between the local terminal and the cloud server and improves communication efficiency. Thus, the response time of the recognition result is greatly shortened.

### Threat model

We illustrate the security threats in the face recognition system based on edge computing from two aspects: user privacy and data reliability.

1. Threats to user privacy. As an untrusted third party, the cloud server is curious about the face data submitted by the edge. In the process of model training or recognition, it may use some intermediate information to further analyze the associated private information. Correspondingly, an attacker can easily obtain facial images, training models, and other associated private information by accessing the cloud server.
2. Threats to data reliability. The local terminal is the source of face data. If it is used by an attacker, the reliability of the data will not be guaranteed. On the one hand, the attacker may submit forged facial data through illegal local terminals; on the other hand, legitimate local terminals may also submit low-quality or invalid facial data (due to the poor objective environment of the image acquisition equipment, the image quality poor, or the user’s maliciousness) will reduce the accuracy of model training, which in turn affects the effectiveness of system functions. In addition, multiple attackers may conspire to send invalid data to disrupt the operation of the system.

### Security goals

In order to effectively solve the user privacy threats and data reliability threats in the system, the designed privacy protection scheme needs to achieve the following security goals:

1. Protect user data privacy. The third-party server or attacker cannot obtain user privacy information. First, the face image data submitted by the edge to the cloud server cannot be raw data, but data processed by privacy protection algorithms. Then submit it to the cloud server for normal model training or recognition so that the system functions are not affected. In addition, a third party or an attacker cannot infer the user’s original face image or related private information through the obtained intermediate information.
2. Ensure data reliability and improve data quality. The edge reviews the authority of the local terminal, and only legal users who have passed the registration can submit data to the edge or receive feedback information from the edge. The edge does not exchange any information with illegal terminals. In addition, the edge preprocesses the image data submitted by the legal local terminal to ensure that the quality of the face image data can meet the needs of model training and recognition.
3. Reduce communication load and improve system response time. Although the data quality of the face image after edge preprocessing is guaranteed, it is still high-dimensional data. If the edge directly submits high-dimensional data, it will put a lot of pressure on network communication, and it will also cause a long system response delay, especially in large-scale data scenarios. Therefore, it is necessary for the edge to perform further dimensionality reduction processing on the face image, reduce the amount of data transmitted between the remote server and itself, reduce the communication burden, and effectively improve the communication efficiency.

## Our proposed framework

**Fig. 2:**
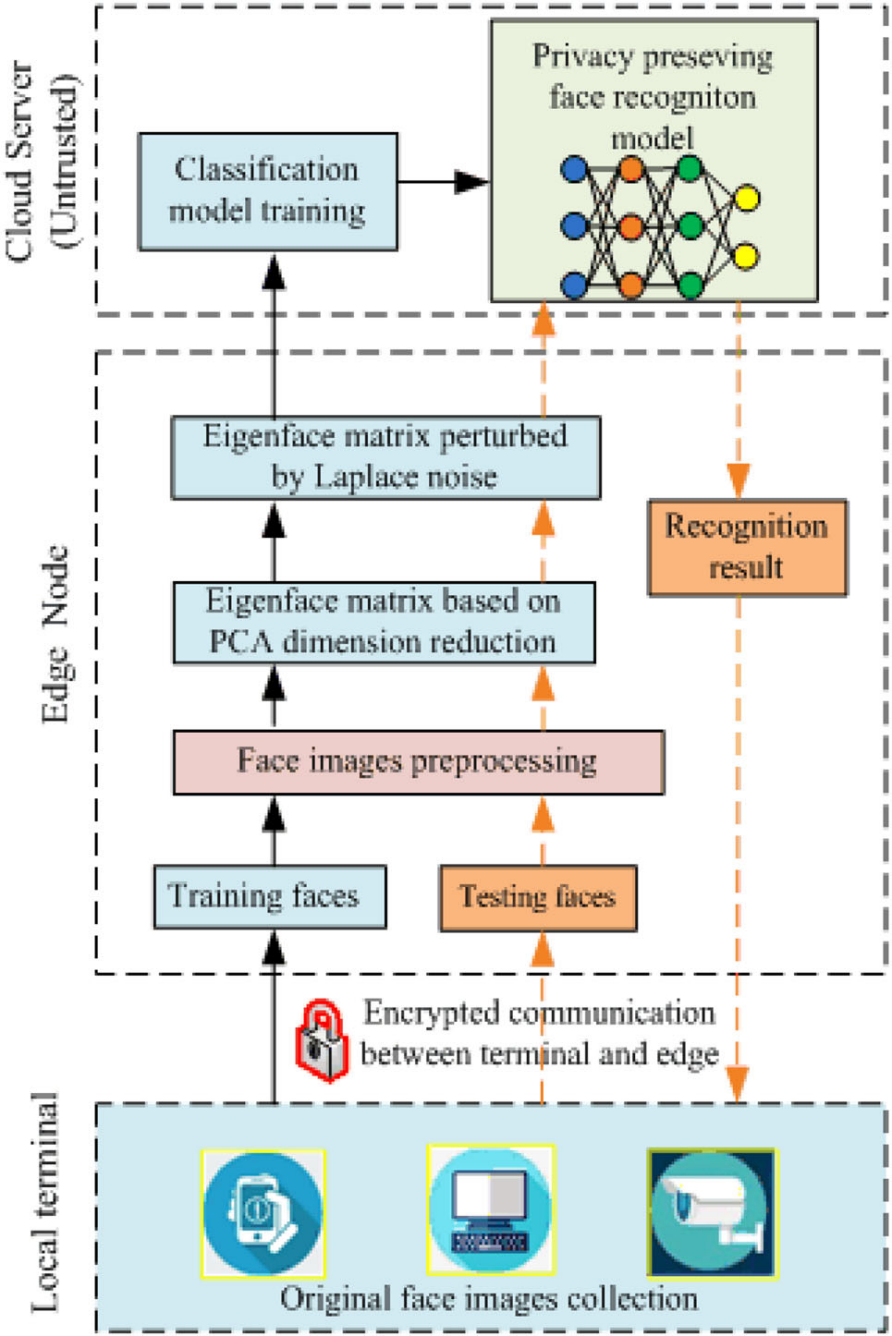
The flowchart of our proposed framework of EFR system

In this section, we discuss the privacy protection framework of the entire face image processing process under the three-level FR system architecture. The flow chart of our framework is shown in Fig. 2.

First, in the data aggregation stage, the terminal device submits the collected face images to the edge through an encrypted communication channel to form a face database. Second, in the model training phase, the edge sequentially performs the following operations on the face image: preprocessing, eigenface generation, and eigenface perturbation. Then, the output perturbed eigenface is sent to the cloud server for classification model training to obtain a privacy-protected recognition model. Finally, in the face recognition stage, the terminal submits the test image, and the edge performs image processing and disturbance according to the above steps and submits the result to the cloud. The cloud uses the trained model to get the recognition results and feed them back to the edge. The terminal device obtains the recognition response from the edge.

Based on the key exchange protocol and LDP technology, we solve the problem of data reliability and user privacy protection throughout the life cycle of face images in recognition processing.

### Encrypted communication and authentication

To prevent attackers from submitting forged data to the system through illegal terminals to achieve the purpose of disrupting the normal operation of the system or stealing key intermediate information. At the same time, to further ensure the quality of the data and improve the reliability of the data, it is necessary to authenticate the local terminal and establish a secure data exchange channel between the local terminal device and the edge node.

The process of establishing secure communication and identity authentication between the terminal device and the edge node based on the key exchange protocol is briefly described as follows:

1. First, the terminal device and the edge node are based on a key exchange protocol, namely the Diffie–Hellman protocol to generate a temporary key. This key is only owned by the edge and the device to ensure subsequent communication security. The temporary key generation process is:
  - The device sends its own $ID_{i}$ to the edge, requesting a temporary key.
  - The edge node randomly selects three numbers *g*, *p*, *a*, where *a* is a private parameter; sends *g*, *p* and $A = ({g^a})\bmod p$ to the device.
  - After the device receives the above data set, it also selects a private parameter *b* and replies $B = ({g^b})\bmod p$ to the edge node as a response. At this time, the device can calculate its temporary key: $K = \left( {{A^b}} \right) \bmod p$, and the edge node also calculates its own temporary key: $X = \left( {{B^a}} \right) \bmod p$. Because of $K = X$, the edge and the device got the same temporary key.
2. The device applies to the edge node for registration.
  - The device encrypts its own $ID_{i}$ with the temporary key *K* and sends it to the edge node as a registration application;
  - After receiving the registration application from the device, the edge node extracts $ID_{i}$ and uses the private key *key* and a one-way hash function *H* with a 64-bit output to generate a registration key for it: $p{w_i} = H(I{D_i},key)$, then sends ${c_1} = X\left( {p{w_i}} \right)$ to the device.
  At this point, the device has completed the registration on the edge network.
3. The edge node authenticates the device.
  - The device receives $pw_{i}$, calculates ${\delta _i} = H\left( {t \oplus p{w_i}} \right)$, $c = \left( {I{D_i},{\delta _i},t} \right)$, and replies ${c_2} = K\left( c \right)$ to the edge node as a response, where *t* is the current system time.
  - The edge node receives feedback from the device at the moment $t^{\prime }$. First, check the validity of $ID_{i}$. If the format is incorrect, the identity authentication will fail. Secondly, determine the validity of the time interval $\Delta t = t^{\prime } - t$ and determine whether it is within the expected effective transmission delay time interval. If it times out, the device will be denied access to the edge network.
  - When both $ID_{i}$ and the time interval $\Delta t$ are valid, the edge node will carry out further calculations:
    7 $$\begin{aligned} p{w_i}= & {} H(I{D_i},key) \end{aligned}$$
    8 $$\begin{aligned} {\delta _i^{\prime }}= & {} H(t \oplus p{w_i}) \end{aligned}$$
    If $\delta _i^{\prime }$ matches with $\delta _i$, the terminal device has passed the identity authentication and can access the edge network, and the edge will send the network key to the terminal device. Otherwise, the edge network rejects the terminal device.
4. The terminal device and the edge node use AES algorithm to realize data encryption communication.
  - The terminal device computes hash value of the original face $D_i$, $h_i=H_1(D_i)$, then sends $K(D_i, h_i)$ to the edge node. Edge node decrypts the ciphertext to obtain face image for subsequent processing(model training or recognition).
  - The edge feeds back the recognition result $X(r_i, h_i^{\prime })$ containing the hash of original face. The terminal device accepts this result $r_i$ only when $h_i$ and $h_i^{\prime }$ are equal.

### Image processing and privacy protection

The collected face image is uploaded to the edge computing node via encrypted communication by the registered terminal device. The edge computing node preprocesses the face image, including correcting the angle of the face to a standardized direction, cutting out redundant data, filter out the interference noise, scale the size of the face image to a predetermined size, etc. Since it is not the focus of this article, the specific process is omitted here.

#### Constructing eigenface

The vector dimension after vectorization of face images is generally high, so face image recognition is a classic high-dimensional small sample problem. PCA can be used to reduce the dimensionality, and the resulting low-dimensional subspace is generally called the “face space”. Dimensionality reduction based on PCA can find a set of basis vectors used to define the “face space”. These vectors can describe the distribution of face images in space.

Assuming that the number of face image samples is *n*, the dimension is *d*, which constitutes an original sample set ${D_{n \times d}}$, and the vector generated by the *i*th face image is recorded as $x_{i}$, then the average image vector of the sample set is:

9 $$\begin{aligned} \mu = \frac{1}{n}\sum \limits _{i = 1}^n {{x_i}} \end{aligned}$$

Then, center the sample data according to $\mu$:

10 $$\begin{aligned} {x_i} = {x_i} - \mu \end{aligned}$$

Further obtain the covariance matrix *A*:

11 $$\begin{aligned} A = \frac{1}{n}\sum \limits _{i = 1}^n {({x_i} - \mu ){{({x_i} - \mu )}^T}} = \frac{1}{n}C{C^T} \end{aligned}$$

Among them, is the set of relative mean image difference of each image:

12 $$\begin{aligned} C = \begin{bmatrix} {{x_1} - \mu },&{{x_2} - \mu },&\cdots ,&{{x_n} - \mu } \end{bmatrix} \end{aligned}$$

Let the non-zero eigenvalue of matrix $CC^{T}$ be ${\lambda _i}(i = 1,2, \ldots ,n)$, $v_{i}$ is the eigenvector corresponding to the non-zero eigenvalue $\lambda _{i}$. The matrix $CC^{T}$ is orthogonalized and normalized, and the calculated eigenvector $u_{i}$ is:

13 $$\begin{aligned} {u_i} = \frac{1}{{\sqrt{{\lambda _i}} }}\sum \limits _{i = 1}^n {C{v_i}} , (i=1,2,\ldots ,n) \end{aligned}$$

The calculated eigenvectors are sorted by size, and the first *k* largest eigenvalues and their corresponding orthogonal normalized vectors $u_{1}, u_{2}, \ldots , u_{k}$ are selected to form the eigenface space. Algorithm 1 describes the basic process of constructing low-dimensional eigenface based on PCA.
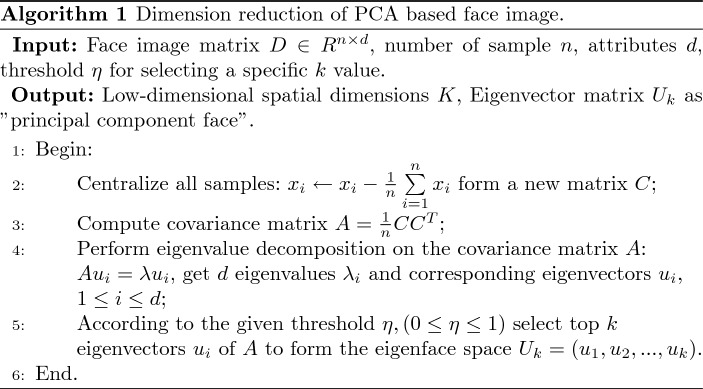


#### Eigenface perturbation algorithm

The face image after PCA dimensionality reduction, that is, the projection matrix is quite different from the original face. However, because the “eigenface” still retains the main feature information that can reflect the corresponding human face, the attacker can completely recover the approximate face image close to the original data through the “eignface”. Therefore, if the edge node directly submits the “characteristic face” to the untrusted server, there will be a greater risk of privacy leakage, not only in the transmission process, but also in the cloud storage, processing, and publishing process. In [17], the authors proposed an output perturbation method based on the Laplacian mechanism. We review the algorithm in the paper, as follows.
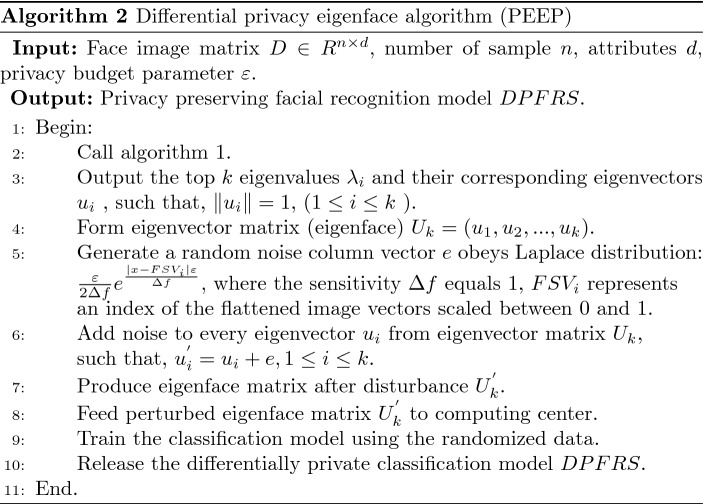


According to the previous analysis, the larger the feature value, the larger the proportion of the information contained in the corresponding component, which means that the information contained in the corresponding component is more important. However, Algorithm 2 (PEEP) [17] adopts equal noise addition for eigenvector matrix elements, which will introduce some risk. On the one hand, the amount of noise introduced will increase with the increase of the number of matrix elements, which reduces the availability of data; On the other hand, adopting consistent equal noise addition, ignoring the difference in the importance of the information contained between the principal components, resulting in excessive loss of privacy budget.

Based on these considerations, we propose a novel LDP budget allocation algorithm for eigenface based on the parallel characteristics and the output disturbance mechanism of DP [36, 40]. The main idea is to adopt DP budget divisions according to the sensitivity and importance of the information contained in the principal components and add different amounts of noise to different column elements of the eigenvector matrix. Algorithm 3, called PEPI, allocates the LDP budget according to the difference in the proportion of principal component feature information.
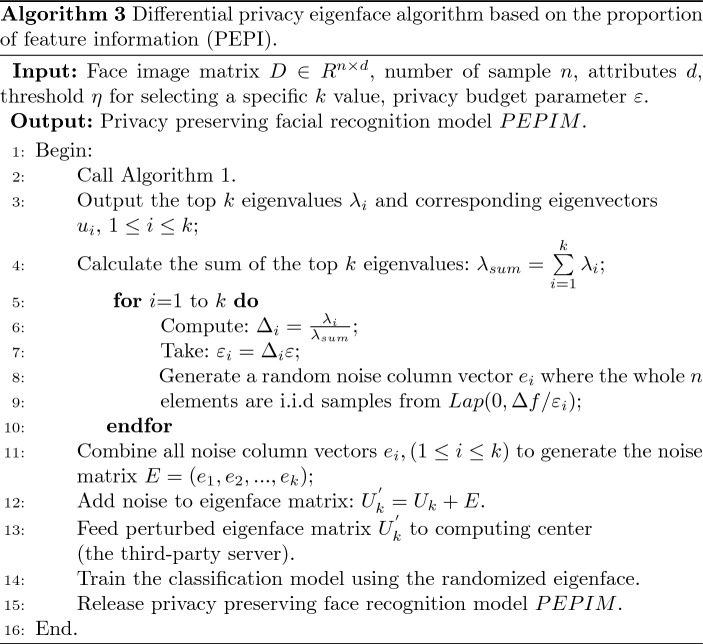


## Theoretical analysis

### PEPI provides $\varepsilon$-LDP protection

#### Theorem 2

In PEPI algorithm, given original data set $D \in {R^{n \times d}}$ and top *k* eigenvectors matrix $U_k$, denote $f\left( D \right) = {U_k}$; then, the sensitivity of the function $f\left( D \right)$ equals $\sqrt{k}$.

#### Proof

In PEPI, the input is a face image, and each face image can be regarded as each vector, which is scaled to the interval [0,1] and then subjected to PCA dimensionality reduction processing. And, a set of feature vectors orthogonal to each other are generated, that is, eigenfaces (select the top *K* with the highest proportion of eigenvalue information). In PEPI, noise is added to these feature vectors. So, the sensitivity of PEPI can understand the maximum difference between eigenvectors. This can be expressed by the equation:

14 $$\begin{aligned} \Delta f = \max \left\{ {{{\left\| {{u_{i}^{\prime }} - {u_i}} \right\| }_1}} \right\} , \end{aligned}$$

As shown in the formula (14), ${u_i}$ represents a flat image vector scaled in the interval [0, 1], and ${u_{i}^{\prime }}$ is adjacent to ${u_i}$,${\left\| {{u_i}} \right\| _2} = 1$, ${\left\| {{u_{i}^{\prime }}} \right\| _2} = 1$, $1 \le i \le k$.

15 $$\begin{aligned} \left\| {{u_{i}^{\prime }} - {u_i}} \right\| _1^2&= \left( \sum \limits _{j = 1}^k {\mid {{u_{i,j}^{\prime }} - {u_{i,j}}} \mid } \right) ^2 \nonumber \\&\le k\sum \limits _{j = 1}^k {\mid {{u_{i,j}^{\prime }} - {u_{i,j}}} \mid } ^2 \nonumber \\&\le k\left\| {{u_{i}^{\prime }} - {u_i}} \right\| _2^2 \nonumber \\&\le k \end{aligned}$$

So, ${\left\| {{u_{i}^{\prime }} - {u_i}} \right\| _1} \le \sqrt{k}$, where *k* is the main component.

At this point, the proof of Theorem 2 is completed. $\square$

#### Theorem 3

Allocating the LDP budget according to the difference in the proportion of principal component feature information satisfies $\varepsilon$-LDP and can provide $\varepsilon$-level privacy protection for face images.

#### Proof

According to Algorithm 3, the expression of the noise eigenface matrix is:

16 $$\begin{aligned} U^{\prime } = U + E , \end{aligned}$$

where $E = \left( {{e_1},{e_2},...,{e_k}} \right)$, ${e_i}(1 \le i \le k)$ means a noise column vector in which the whole *n* elements are i.i.d samples from $Lap(0,\varepsilon _i /\Delta f)$.

Let ${D_{n \times d}}$ and $D_{n \times d}^{\prime }$ are two arbitrary neighbor matrices, only one data record, that is, one vector is different, for example $D_{i}$ and $D^{\prime }_{i}$, $1\le i\le n$. There is a mapping relationship: $f:D \rightarrow {U_k}$, such that, $f(D) = {U_k} = ({u_1},{u_2},...{u_k})$, and $f(D^{\prime }) = {U_{k}^{\prime }} = ({u_{1}^{\prime }},{u_{2}^{\prime }},...{u_{k}^{\prime }})$. The disturbance output results *O* on *D* and $D^{\prime }$ can be expressed as: $O = A(D) = f(D) + \left( {La{p_1}\left( {\frac{{\Delta f}}{{{\varepsilon _1}}}} \right) ,La{p_2}\left( {\frac{{\Delta f}}{{{\varepsilon _2}}}} \right) ,...,La{p_k}\left( {\frac{{\Delta f}}{{{\varepsilon _k}}}} \right) } \right)$. In order to simplify the following description, we remember the output vector $O = ({y_1},{y_2},...,{y_k})$.

Then there is:

17 $$\begin{aligned} \Pr [A(D) = O]= & {} \prod \limits _{i = 1}^d {\frac{{{\varepsilon _i}}}{{2\Delta f}}} {e^{ - \frac{{{\varepsilon _i}}}{{\Delta f}}\mid {{u_i} - {y_i}} \mid }} \end{aligned}$$

18 $$\begin{aligned} \Pr [A(D^{\prime }) = O]= & {} \prod \limits _{i = 1}^d {\frac{{{\varepsilon _i}}}{{2\Delta f}}} {e^{ - \frac{{{\varepsilon _i}}}{{\Delta f}}\mid {{u_{i}^{\prime }} - {y_i}} \mid }} \end{aligned}$$

So,we can further get:

19 $$\begin{aligned} \frac{\Pr [A(D) = O]}{\Pr [A(D^{\prime }) = O]}&= \frac{\prod \limits _{i = 1}^k {{\frac{\varepsilon _i}{\Delta f}}{e^{{-\frac{\varepsilon _i}{\Delta f}}\mid {u_i} - {y_i} \mid }}}}{\prod \limits _{i = 1}^k {{\frac{\varepsilon _i}{\Delta f}}{e^{{-\frac{\varepsilon _i}{\Delta f}}\mid {u_i^{\prime }} - {y_i} \mid }}}} \nonumber \\&= \prod \limits _{i = 1}^k {e^{ {- \frac{\varepsilon _i}{\Delta f}}\left( {\mid {{u_i} - {y_i}} \mid - \mid {{u_i^{\prime }} - {y_i}} \mid } \right) }} \nonumber \\&= e^{\frac{\varepsilon }{\Delta f}\sum \limits _{i = 1}^k {\left( {\mid {{u_i^{\prime }} - {y_i}} \mid - \mid {{u_i} - {y_i}} \mid } \right) }} \end{aligned}$$

However, for each $\mid {{u_{i}^{\prime }} - {y_i}} \mid - \mid {{u_i} - {y_i}} \mid$, we regard $y_i$ as a variable. According to the absolute value inequality, the following relationship exists:

20 $$\begin{aligned} \sum \limits _{i = 1}^k {\left( {\mid {{u_i^{\prime }} - {y_i}} \mid - \mid {{u_i} - {y_i}} \mid } \right) }&\le \sum \limits _{i = 1}^k {\mid {u_i^{\prime }} - {u_i} \mid } \nonumber \\&\le {\mathop {\max }\limits _{D,{D^{\prime }}}} \left( \sum \limits _{i = 1}^k {\mid {u_i^{\prime }} - {u_i} \mid } \right) \nonumber \\&= \Delta f \end{aligned}$$

In the end,we get:

21 $$\begin{aligned} {\frac{{\Pr [A(D) = O]}}{{\Pr [A(D^{\prime }) = O]}}} \le {e^{\varepsilon } } \end{aligned}$$

This completes the proof of Theorem 3. $\square$

#### Theorem 4

PEPI (Algorithm 3) provides $\varepsilon$-LDP protection for output.

#### Proof

The training of classification models based on perturbed eigenfaces can be seen as an independent transformation process for the output of $\varepsilon$-LDP (Theorem 3). Since DP has the property of post-processing immunity, which means that the model training result (PEPIM) and the model testing result (recognition result) are still protected by DP.

Thereby, the PEPI (Algorithm 3) satisfies $\varepsilon$-LDP, which completes the proof of Theorem 4. $\square$

### System security analysis

The system considers the actual application scenarios, and does not assume that the cloud server is completely credible as a third party. Moreover, as a shared storage space, there are other resource users and visitors, and it is not ruled out that individual attackers want to access the image data information in the cloud space and obtain valuable information from it. The system can ensure the safety of the following aspects:

1. The transmission of terminal data to the edge is secure.
  - The temporary key provides a guarantee for the establishment of secure communication.
  - A terminal device that wants to access the edge network must register by submitting its own ID in order to obtain a legal identity. Then, in the data preprocessing stage, once the edge node finds that a certain device submits fake data, it immediately prohibits its access, refuses to provide it with subsequent functional services, and conducts identity tracking. In this way, it can resist poisoning attacks and collusion attacks.
  - In the identity authentication phase, the edge node judges the validity of the response time from the terminal device. If it times out, even if the ID and registration key provided by the device are correct, it will still be rejected by the edge node. In this way, the terminal device’s replay attack on the system is avoided.
  - The encrypted transmission based on the AES algorithm further ensures the security of data communication between the terminal device and the edge node.
2. During the transmission process from the edge node to the cloud platform, the transmission data stolen by the attacker is the principal component characteristic data protected by LDP, so that the attacker cannot directly obtain and infer the original face image data. Therefore, this process can ensure that the face image data is safe.
3. User privacy is guaranteed because the edge node submits a face feature data set that meets differential privacy protection, and does not carry any other identifying information associated with the user terminal device, such as *ID* or *location*. Therefore, even if the cloud server leaks relevant information in the face image processing, the attacker cannot associate the user with the leaked information.

## Experimental

### Data set

We choose two open source datasets to test the performance of the algorithm, namely a small-scale face image dataset named fetch−olivetti−faces (Olivetti) and a larger-scale face image dataset named lfw−funneled people (LFW). The Olivetti data set is taken by the Cambridge Laboratory in the UK and contains photos of 40 different individuals. The LFW data set is a database compiled by the Computer Vision Laboratory of Massachusetts State University Amherst, and is usually used to study face recognition problems in unrestricted environments. LFW contains more than 13,000 gray-scale face images collecting on the Internet.

#### Feature extraction

**Fig. 3:**
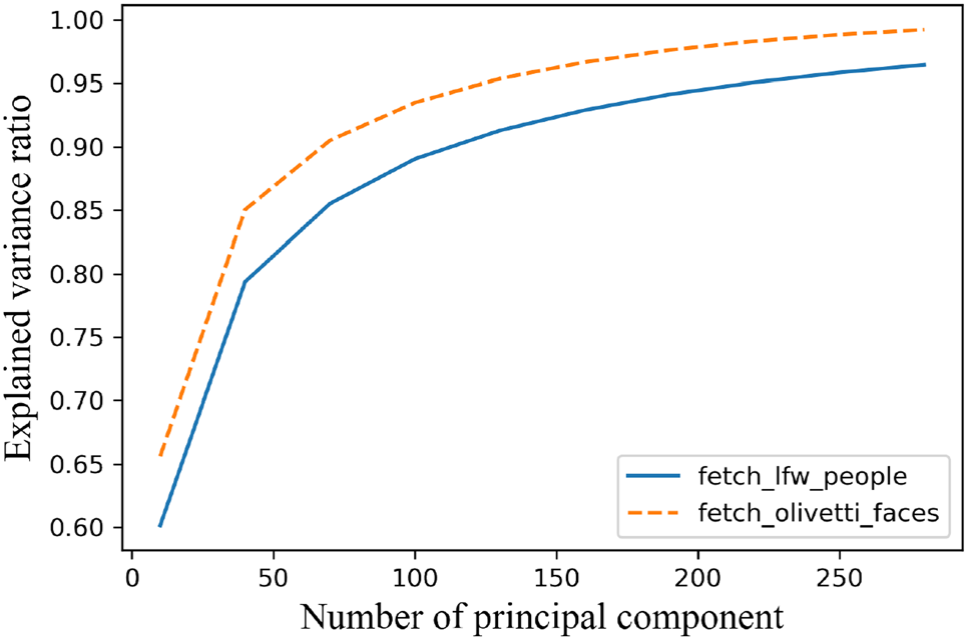
Explained variance ratio varies with *K*

We use PCA to reduce the dimensionality of high-dimensional images, which can drop the complexity of subsequent image processing while maximizing the preservation of the original information. Of course, the more features are retained at the end, the less information is lost, but the computational complexity is higher. We implement parameters tuning using the accumulative contribution threshold $\eta$. Figure 3 shows the cumulative explained variance ratio with the number of principal components *K*.

**Fig. 4:**
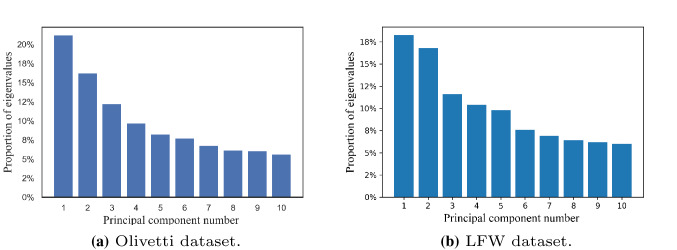
The proportion of eigenvalues of the top 10 principal components

**Fig. 5:**
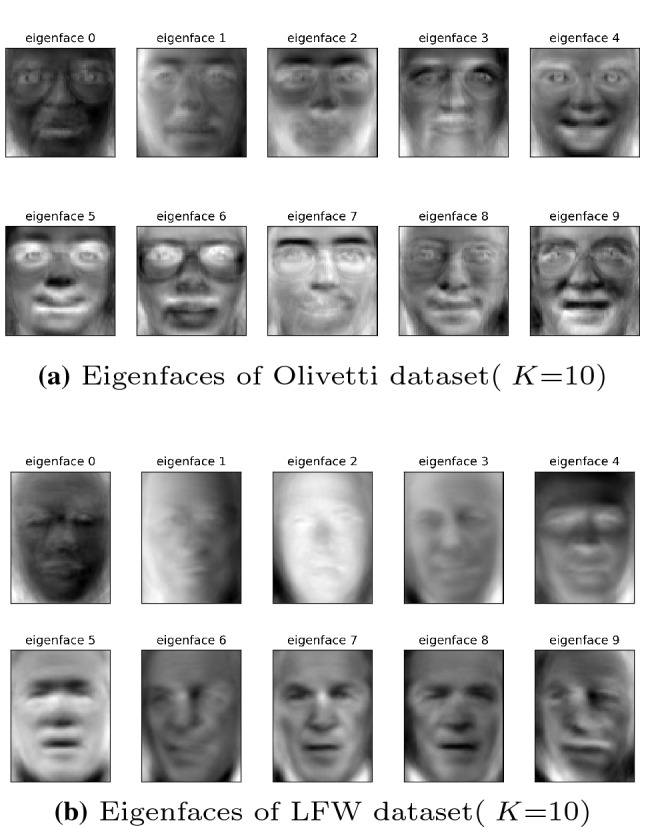
Part of the sample images in the source input data set (the training set used to build the PCA model) are processed for dimensionality reduction, and only a set of eigenfaces generated by the first 10 most important eigenvalues are retained

Figure 4 shows the proportion of the eigenvalues of the top 10 principal components in their respective data sets. Usually, we select the first *K* eigenvectors corresponding to the eigenvalues to describe the original face space. This set of orthogonal vectors is called eigenface. Eigenface shows the basic features of the input image, and any face image is a combination of a group of eigenface. Figure 5 shows the eigenface belonging to the above two data sets when K=10.

#### Eigenface disturbance

**Fig. 6:**
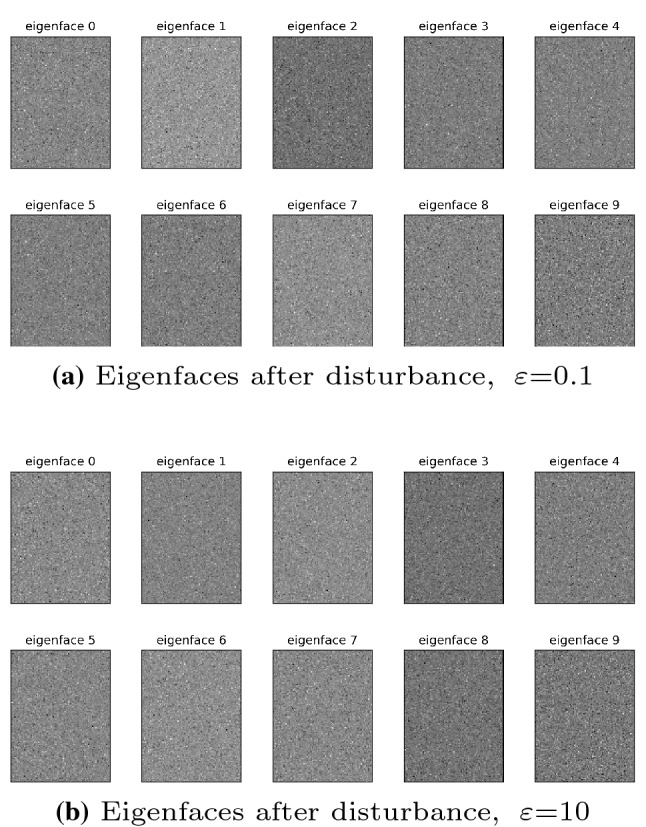
Perturb the eigenface (LFW dataset), $\varepsilon$ = 0.1, 10. The smaller the privacy budget $\varepsilon$, the more obscure the feature information

The eigenface hides the most important biological characteristics of the face database, and with the help of effective face reconstruction technology, the original face image information can be restored through the eigenface. Therefore, it is necessary to protect the privacy of eigenfaces. Figure 6 shows the eigenface (Fig. 5b) after implementing the PEPI algorithm to add noise perturbation (privacy budget $\varepsilon = 0.1$). As shown in the figure, at this time, it is no longer possible to detect any biological features of the face image from the eigenface with naked eyes. Even in the most relaxed situation (privacy budget $\varepsilon = 10$), the disturbed feature face still cannot be observed by naked eyes, as shown in Fig. 6b.

### Model training and recognition

We use Tensorflow environment and Python language programming for simulation experiments. The server used has the following resource configuration: CPU Intel Xeon CPU E5-2620 v4 2.10 GHz, Hynix DDR4 16 G memory, hard disk with 300 G (solid state) plus 2 T (mechanical). The recognition performance test algorithm uses support vector machine (SVM) to perform multi-class recognition of face images after PCA dimensionality reduction.

#### Performance variance with respect to $\varepsilon$

**Fig. 7:**
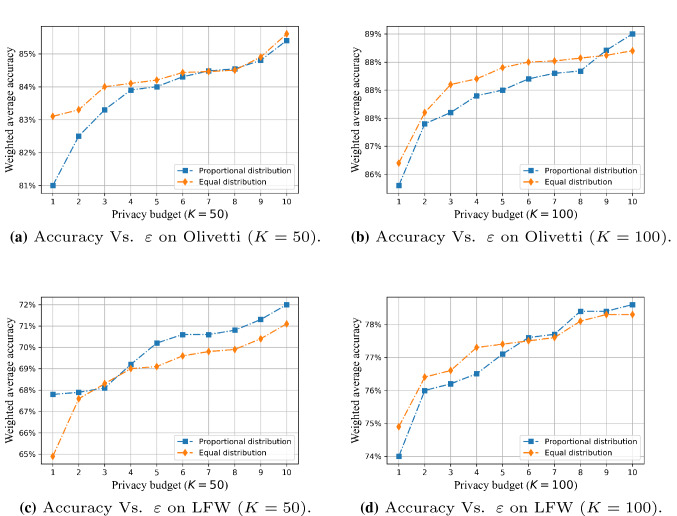
Comparison of FR performance between PEPI (privacy budget proportional distribution) and PEEP (privacy budget equal distribution) on two dataset, $K=50,100$

**Fig. 8:**
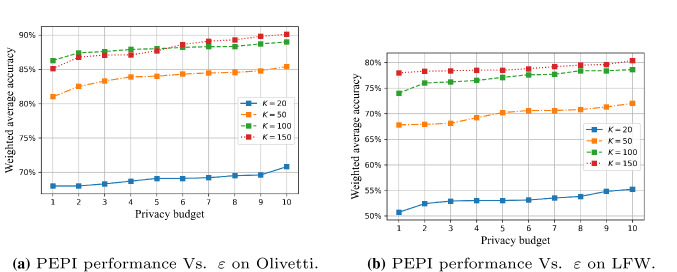
The FR performance of the PEPI changes with the privacy budget $\varepsilon$ when the principal component *K* takes four different values: 20, 40, 100 and 150

We used the weighted average of training accuracy, recall, and F1 scores to reflect the impact of different levels of privacy budget $\varepsilon$ on the performance of the algorithm in two data sets, and plotted the data, as shown in Figs. 7 and 8.

Figure 7 shows the comparison between the PEPI algorithm proposed in this paper and the existing PEEP algorithm [17]. It can be seen from the figure that when the number of principal components *K* is constant, increasing the privacy budget improves the accuracy, because a higher privacy budget imposes less randomized disturbance on the eigenface. In addition, when $1<\varepsilon <4$, the PEEP algorithm that allocates the privacy budget by the ratio of information can obtain a slightly higher accuracy rate than the PEPI algorithm that allocates the privacy budget based on the information ratio. When $\varepsilon >4$, as the privacy budget increases, the performance of the PEPI algorithm is closer to or even surpasses the performance of the PEEP algorithm.

Figure 8 shows the changes in the performance of the PEPI algorithm with the privacy budget when the *K* values are 20, 50, 100, and 150. Through comparison, we find that the number of principal components that retain the original image data after dimensionality reduction also directly affects the accuracy of the final classification. The smaller the *K* value, the less the proportion of original image feature information is retained, which will restrict the performance of later image analysis to a certain extent.

However, when the *K* value reaches a certain value (for example, 100) and the reduced image can cover more than 95% of the feature information of the original image, the performance of the algorithm will be less affected by changes in privacy budget. There is almost no difference in the amount of information contained in $K=150$ and $K=100$ images for Olivetti. However, difference lies in the privacy budget allocated to each retained feature. When the total privacy budget is the same, the privacy budget allocated to each feature of K=150 is smaller than that of $K=100$. The smaller the privacy budget allocated to each feature, the stronger added noise perturbation. This leads to $K=100$ behaves better than $K=150$ for the lower privacy budgets, as shown in Fig. 8a.

#### Performance variance with respect to *K*

PCA retains the main feature components, and the value of the principal components *K* directly affects the proportion of these feature components that contain the original complete information, which in turn affects the accuracy of face recognition. We keep the privacy budget $\varepsilon$ at a fixed value (for example, 0.5, 1, 5, 10, 100), only change the number of principal components *K*, and then compare the accuracy of face recognition in three different situations: no privacy protection, equal allocate privacy budgets and allocate privacy budgets based on the proportion of feature information.

**Fig. 9:**
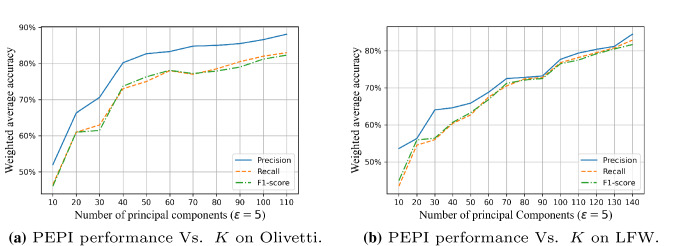
The FR performance of the PEPI changes with the number of principal components *K* when $\varepsilon =5$

Figure 9 shows that the face recognition performance of the PEPI algorithm changes with the principal component when $\varepsilon =5$ through three indicators of weighted average accuracy: precision, recall, and F1-score. As shown in the figure, when the privacy budget is constant, the overall trend of the accuracy is significantly improved with the increase of the value of the principal component. However, when *K* reaches a certain size, the increase in accuracy will tend to be flat.

**Fig. 10:**
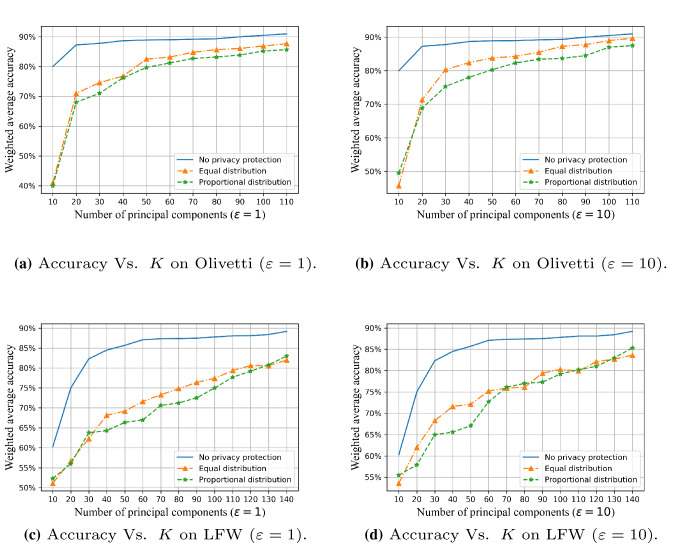
Comparison of the accuracy of FR in three different situations: no privacy protection, PEPI (privacy budget proportional distribution) and PEEP (privacy budget equal distribution)

Figure 10 shows the classification accuracy on the data set processed by the PEPI algorithm when $\varepsilon = 1, 10$, and compares it with the two cases: no disturbance and PEEP. As shown in the figure, when the value of *K* increases from 10 to 20, the performance improves rapidly. After *K* increases to 20, the performance gradually improves. This is because the first 20–40 principal components represent the most important features of the original face image. Although the performance improvement is not too obvious when *K* is a value after 40, the overall performance is improved, which also shows that the more principal components retained, the better the performance.

**Fig. 11:**
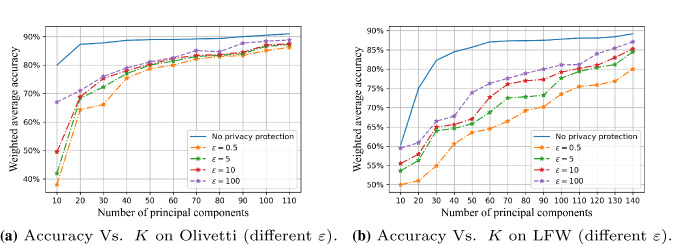
The FR performance varies with the number of principal components *K* under different values of $\varepsilon$. Here is a comparison of the difference in performance when the privacy budget $\varepsilon$ takes four different values: 0.5, 5, 10, and 100

In addition, we also compared the changes of face recognition performance with principal components under different $\varepsilon$ values (three conventional values: 0.5, 5, 10, and an extreme value: 100), as shown in Fig. 11. It can be seen that the larger the privacy budget and the number of principal components, the better the performance.

### Security comparison

Table 1 shows the security comparison of related privacy-preserving face recognition schemes. The commonality of each scheme is that face recognition is processed in the cloud. The difference lies in the privacy protection method and whether the eigenface is private for the cloud server. Outsourcing can reduce the computational complexity for face image owners and authenticated users. Since eigenfaces still contain some private information, privacy needs to be protected before outsourcing. In [13, 14, 18], a homomorphic cryptosystem is utilized to protect the privacy of faces. The projections of the three schemes are all processed in the cloud, but none of the eigenfaces are private to the cloud server. Camara et al. [17] uses LDP technology to achieve eigenface perturbation, however, data poisoning attacks and tampering attacks by malicious users are not addressed.

**Table 1:** Security comparison of related privacy-preserving FR schemes

References	Methods	Eigenface	Defensible attack			
			Data poisoning	Differential	Man-in-the-middle	Tamper
Erkin et al. [13]	PH cryptosystem	Non-privacy	$\checkmark$			
Sadehi et al. [18]	PH cryptosystem	Non-privacy	$\checkmark$			$\checkmark$
Xiang et al. [14]	FH cryptosystem	Non-privacy	$\checkmark$		$\checkmark$	
Chamikara et al. [17]	LDP	Privacy		$\checkmark$	$\checkmark$	
The proposed	LDP, authentication	Privacy	$\checkmark$	$\checkmark$	$\checkmark$	$\checkmark$

## Conclusions

We propose a user privacy protection scheme suitable for FR application scenarios based on EC. This framework takes into account the privacy protection issues during the entire cycle from collection to recognition of face images. A new mechanism of PEPI using the characteristics of LDP is proposed, which combines the proportion of principal component feature information to perform data disturbance to realize the privacy protection of FR users.

Our scheme does not require a trusted third party. The edge center uses localized processing methods to apply randomization before the image reaches the untrusted server. In addition, adjusting the privacy budget parameters according to the proportion of principal component feature information can achieve the maximum balance of privacy protection and data utility compared with existing solutions. Of course, there are still some issues worthy of further research in the future, such as improving the security of the key exchange process, edge-based processing with private multi-dimensional classification information and heterogeneous data.

## Data Availability

The data and material that support the findings of this study are available from the corresponding author upon reasonable request.

## Code availability

The code of this study are available from the corresponding author upon reasonable request.

## Declarations

### Research invovling human and/or animal participants

This study does not involve human participants and/or animal research, which is used for non-life science journals.
